# Faces in Places: Humans and Machines Make Similar Face Detection Errors

**DOI:** 10.1371/journal.pone.0025373

**Published:** 2011-10-05

**Authors:** Bernard Marius 't Hart, Tilman Gerrit Jakob Abresch, Wolfgang Einhäuser

**Affiliations:** Neurophysics, Philipps University Marburg, Marburg, Germany; University of British Columbia, United States of America

## Abstract

The human visual system seems to be particularly efficient at detecting faces. This efficiency sometimes comes at the cost of wrongfully seeing faces in arbitrary patterns, including famous examples such as a rock configuration on Mars or a toast's roast patterns. In machine vision, face detection has made considerable progress and has become a standard feature of many digital cameras. The arguably most wide-spread algorithm for such applications (“Viola-Jones” algorithm) achieves high detection rates at high computational efficiency. To what extent do the patterns that the algorithm mistakenly classifies as faces also fool humans? We selected three kinds of stimuli from real-life, first-person perspective movies based on the algorithm's output: correct detections (“real faces”), false positives (“illusory faces”) and correctly rejected locations (“non faces”). Observers were shown pairs of these for 20 ms and had to direct their gaze to the location of the face. We found that illusory faces were mistaken for faces more frequently than non faces. In addition, rotation of the real face yielded more errors, while rotation of the illusory face yielded fewer errors. Using colored stimuli increases overall performance, but does not change the pattern of results. When replacing the eye movement by a manual response, however, the preference for illusory faces over non faces disappeared. Taken together, our data show that humans make similar face-detection errors as the Viola-Jones algorithm, when directing their gaze to briefly presented stimuli. In particular, the relative spatial arrangement of oriented filters seems of relevance. This suggests that efficient face detection in humans is likely to be pre-attentive and based on rather simple features as those encoded in the early visual system.

## Introduction

To primate vision, faces appear to be a special category of stimuli, processed faster than other stimuli [1], [2] and in specific brain areas [3], [4]. This preferential processing of faces starts from an early age [5] and develops into adolescence [6], [7]. Face processing is of relevance for providing information in highly social environments. For example, behavior towards others is affected by the others' emotional expressions [8] and history of cooperative behavior of others affects face recognition [9]. The first step in face processing lies in reliable and efficient face detection.

Several lines of psychophysical evidence for a special role of faces in human visual detection exist. First, although object classification is remarkably fast, faces can be detected faster than other object classes [1]. Second, research in the realm of attention suggests that human face detection may happen pre-attentively [10], although the implications of this result have remained disputed [11]–[13]. Ultra-rapid and pre-attentive face processing implies either an early locus for face detection in the visual cortical hierarchy or the involvement of subcortical structures [1]. This motivates the question, whether a seemingly complex computation like face detection could be performed based on the output of rather simple filters, such as the orientation-selective cells in primary visual cortex (V1).

In machine vision, face detection is a highly successful area. Some face-detection algorithms are so efficient, in terms of both speed and accuracy, they end up in consumer products [14]. The success of this approach is remarkable given that the algorithm only uses the output of a relatively small set of Haar features. Haar features are rectangular filters, which only take binary values (+1 and −1) such that the filter output is computed as sum over one area (akin to an “on” region in physiology) minus the sum over the other area (“off” region) allowing the algorithm to reach high computationally efficiency. By their rectangular structure, the Haar filters predominant in the Viola-Jones algorithm have a pronounced orientation preference. In addition, Haar filters are localized in the sense that their extension is limited to a finite area, which is typically small compared to the size of the image being processed. Differences in spatial frequency characteristics notwithstanding, these properties of localization and orientation selectivity are reminiscent of the defining features of V1 cells [15]. Evidence for the hypothesis that human vision employs similar mechanisms for face detection as the Viola-Jones algorithm will therefore also provide a potential mechanism for face detection by early visual areas. To test this hypothesis, we investigate whether human observers make similar errors as the Viola-Jones algorithm, when asked to direct their gaze to a face. More precisely, in a first experiment we test whether observers mistake Viola-Jones false positives (“illusory faces”) more frequently for real faces than matched stimuli that are correctly classified as non faces. In a second experiment, we test the effect of color and rotation on human face detection by using performance-matched subsets of illusory and real faces. In a third experiment, we test whether a manual response (button press) instead of gaze allocation yields different results. The first experiment shows that illusory faces fool human and machine face detection alike, suggesting that humans use similar features as the Viola Jones algorithm for rapid face detection. The second experiment demonstrates that humans indeed use oriented, localized features for face detection, but also benefit from the presence of color. The third experiment shows that the similarity in errors between the Viola-Jones algorithm and humans is restricted to gaze allocation and does not occur for a manual response, indicating that Viola-Jones-like face processing is restricted to pre-attentive detection.

## Results

We used three categories of stimuli, which were extracted from a central region of natural first-person perspective videos [16]: 1) real faces, locations correctly detected by the Viola-Jones algorithm 2) illusory faces, false positives of the Viola-Jones algorithm, and 3) non faces, correctly rejected locations that were otherwise matched to the illusory face locations (see Methods for details).

### Experiment 1

**Figure 1:**
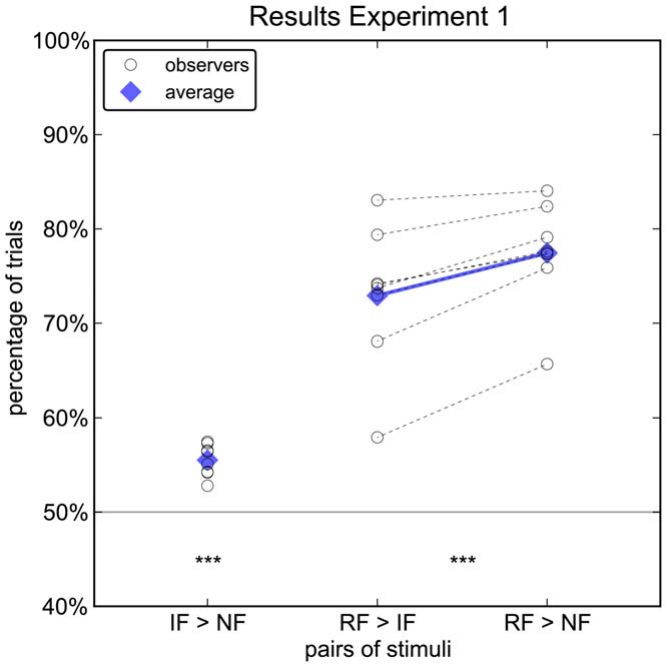
Results of Experiment 1. *From left to right:* percentage of trials in which illusory faces (IF) are selected over non faces (NF), real faces (RF) over illusory faces, and real faces over non faces in experiment 1. *Black circles:* individual observers (500 trials per observer in each condition); *blue squares:* mean over all 8 observers. IFs are selected over NFs above chance by all observers and significantly above chance on average; all observers select fewer RFs over IFs than over NFs, and significantly so on average.

In each trial of experiment 1, observers were presented a pair of stimuli for 20 ms. Each pair consisted of stimuli from two different categories yielding three different types of trials (real face vs. illusory face, real face vs. non face, illusory face vs. non face). We asked observers to make an eye movement to the side where the face had been presented. For pairs of illusory faces and non faces, all 8 observers chose illusory faces more often (55.5%±1.6% (s.d.), range: 52.8%–57.5%, figure 1), a fraction significantly larger than the chance level of 50% (*t*(7) = 9.280, *p*<.001). In addition, all observers chose the real face more frequently when paired with a non face (77.4%±5.1%) than when paired with an illusory face (72.9%±7.0%), and this difference is significant (*t*(7) = 5.382, *p*<.001, paired t-test; figure 1). Hence illusory faces were mistaken for real faces more often than non faces and illusory faces distracted from real faces more efficiently than non faces. This demonstrates that illusory faces as defined by the Viola-Jones algorithm also more likely evoke the illusion of a face for human observers. This similarity of errors provides the first evidence that human face detection and the Viola-Jones algorithm share some computational principles.

**Figure 2:**
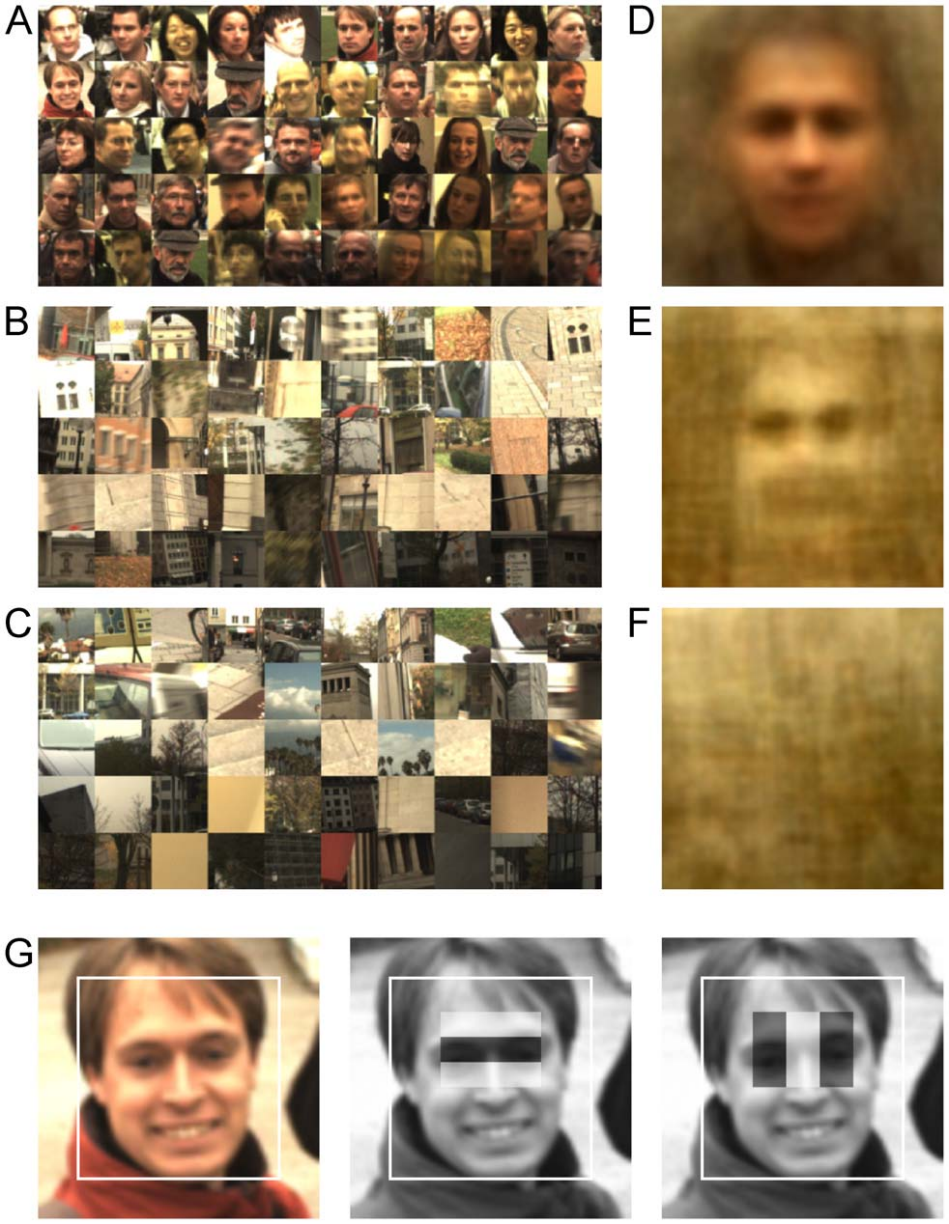
Stimulus Ranking and Average. **A**) Real face stimuli sorted by the probability of being correctly selected in experiment 1; middle row stimuli used in Experiment 2 **B**) illusory face stimuli sorted by the probability of being selected as face in experiment 1; middle row stimuli used in experiment 2 **C**) non face stimuli sorted by the probability of being selected as face in experiment 1. **D**) average of real face stimuli, **E**) average of illusory face stimuli, **F**) average of non face stimuli; averages in panel D–F are computed as pixelwise arithmetic means for each color channel, for an illustration how this average yields the face-like appearance in panel E (figure S1); **G**) real face stimulus with first two Haar filters of the OpenCV implementation of the Viola-Jones algorithm overlaid. The white squares indicate the area designated a face by the algorithm.

In addition to a difference in the probability of being selected as face across categories (real, illusory or non face), there was also substantial variation within categories. Sorting the stimuli of each category by this selection probability (figure 2a–c) suggested that low-level features may in part be responsible for this difference. Indeed, luminance and RMS luminance contrast correlated positively with the probability of being selected as face. These correlations were significant for all categories in combination with any other category (all *p*<.009). Importantly, however, illusory faces and non faces did not differ in luminance (t(97.924) = 1.1961, *p* = .235) or luminance contrast (t(93.099) = 0.4228, *p* = .673). Hence the main result that human observers mistake illusory faces more often for faces than non faces could not be explained by these low-level features.

### Experiment 2

Given that the Viola-Jones algorithm and human face detection used similar features, what were those? A first intuition was generated by averaging the 50 real faces (figure 2d), 50 illusory faces (figure 2e; figure S1) and 50 non faces (figure 2f). In the average real face eyes, nose, mouth and hairline were clearly identifiable. The average illusory face still suggested locations of eye, mouth, nose and face outline, which were all absent in the average non face. Unlike the average real face, which is pinkish, neither illusory nor non-faces distinguished themselves from their background in color. Noting that the Viola-Jones algorithm used localized oriented filters but no color information (figure 2g), we hypothesized that the commonalities of Viola-Jones and human face detection errors had arisen from the spatial arrangement, while color presented a feature available to humans in addition.

To test this hypothesis, we selected ten real face and ten illusory face stimuli, which had had an intermediate probability of being selected as face in experiment 1 (middle row in figure 2a and b). In each trial of experiment 2 observers were presented a pair of one illusory and one real face, each of which was either shown upright or rotated by 90° counterclockwise. In one half of the experiment stimuli were in color, in the other half, stimuli were presented in grayscale (order counterbalanced over the 8 observers). In all other respects, experiment 2 was identical to experiment 1 and 8 new observers participated.

**Figure 3:**
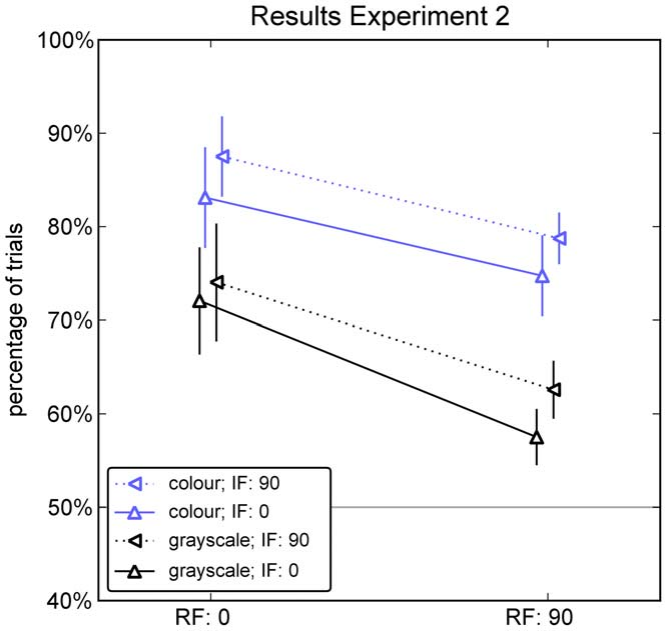
Results of Experiment 2. Correct detection of real faces, when paired with illusory faces (% of trials). Both stimuli could be either upright or rotated (90°), resulting in a 2×2 design. The x-axis denotes the orientation of the real face, the marker the orientation of the illusory face. *Black:* experiment with grayscale stimuli, *Blue:* with colored stimuli. Error bars denote standard errors of the mean over 8 observers, who each performed 100 trials of each type. Real faces are detected better when upright than when rotated in any condition (all data are higher on the left than on the right); illusory faces are better distractors when presented upright (solid lines are always below dotted lines, i.e. real faces are detected worse, when the distractor is upright); performance is better (illusory faces are less frequently mistaken for real faces) for colored stimuli than for grayscale stimuli (blue datapoints consistently above black datapoints).

A three way, repeated measures ANOVA on the probability to select the real face in experiment 2 (figure 3) showed main effects of the orientation of real faces (*F*(1,7) = 17.467, *p* = .004), the orientation of illusory faces (*F*(1,7) = 10.469, *p* = .014) and color (*F*(1,7) = 17.805, *p* = .004). None of the interactions were significant (all *p*>.133). If the real face was rotated, it was selected less frequently (performance decreased); if the illusory face was rotated, it also was selected less frequently (i.e., the real face was selected more often, yielding an increase in performance). If stimuli were colored, the real face was selected more frequently than in any grayscale condition, but the effect of rotation prevailed (figure 3). Hence, irrespective of whether the additional cue of color was available, human face detection was sensitive to the orientation of the stimulus features. Remarkably, this orientation sensitivity also held for illusory faces, indicating that the spatial arrangement of oriented features determined face-like appearance.

### Experiment 3

**Figure 4:**
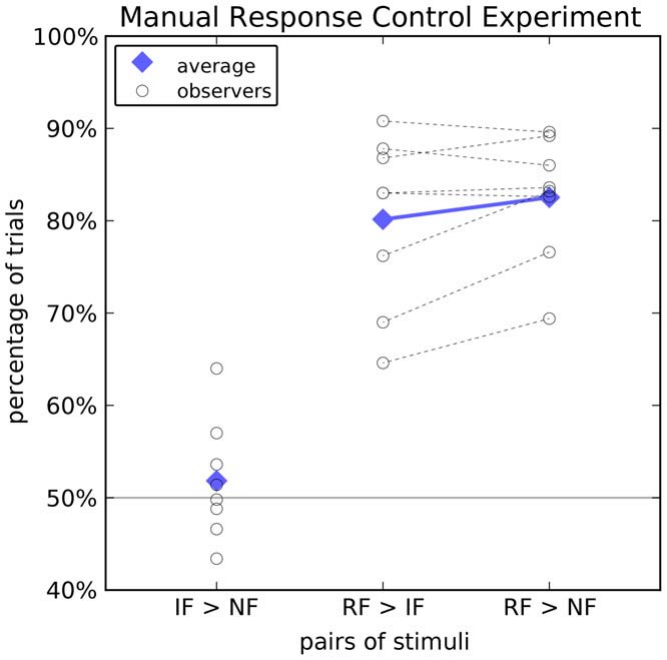
Results of Experiment 3. *From left to right:* percentage of trials in which illusory faces (IF) are selected over non faces (NF), real faces (RF) over illusory faces, and real faces over non faces in experiment 3 (report with manual response). Notation and markers as in figure 1 - black circles: individual observers; blue squares: mean over all 8 observers. Illusory faces are neither preferred over non faces nor are illusory faces mistaken more frequently for real faces than are non faces. The effects observed in experiment 1 with eye-movement responses (figure 1) are thus absent for the manual response used here.

Using an eye-movement as response was motivated by earlier studies on ultra-rapid face detection [1], [2]. To assess whether this mode of response is critical for the obtained results, we asked 8 new participants to perform the task of experiment 1, using a manual response (button press) instead of an eye movement to indicate the side the face had been on. Unlike in experiment 1, we find no preference for illusory faces over non-face stimuli (51.8%±6.4%, range: 43.4%–64.0%; comparison to chance level: t(7) = 0.80, p = 0.45; figure 4). Similarly, the number of correct trials for real faces when paired with illusory faces (80.2%±9.4%, range: 64.6%–90.8%) was statistically indistinguishable from pairing with non faces (82.5%±6.7%, range: 69.4%–89.6%,t(7) = 1.82,p = 0.11). Hence, only gaze direction, but not the manual response are subject to the same error patterns as the Viola-Jones algorithm, indicating that gaze is driven by a different mode of face processing than manual responses; a mode that is presumably pre-attentive.

## Discussion

In the past, face classification algorithms have often benefited from the measurement of human error patterns [17]. Here, we in turn use the similarities between human and machine vision to gain insight into human face detection. Our experiments demonstrate that patterns mistaken for faces by a standard computer vision algorithm frequently fool human rapid face detection as well. Together with the sensitivity of both man and machine to rotation of these patterns, this suggests that human rapid face detection relies on simple features: localized and oriented luminance transitions.

As evidenced by the above chance selection of faces over illusory faces for gaze direction, humans use additional cues for face detection beyond the features of the Viola-Jones algorithm. These might become increasingly relevant for longer viewing times: during prolonged viewing, none of the illusory faces would be mistaken for a real face irrespective of response mode. We indeed show that color is such a feature, which increases performance already at our short presentation times. However, color does not fully override the information by oriented features as the general pattern of results prevails (figure 3). This indicates that Viola-Jones features are particularly useful when other features are not (yet) available, i.e. in a pre-attentive mode of processing.

In the present study we consider false positives of the Viola-Jones algorithm as compared to matched non face stimuli. Alternatively, one could consider false negatives (misses) and compare performance on these stimuli to true positives (real faces). Since the Viola-Jones algorithm in its default configuration with default training set is rather liberal, misses seem substantially rarer than false positives and are often related to partial occlusions, shadows, small size or non-frontal views. Consequently, detecting misses unambiguously can also be challenging for human observers, such that the definition of a miss will not be free of annotator bias (note that for misses the entire video material would have to be screened manually, while for false alarms only frames with detection need to be inspected). To mimic a situation as natural as possible, we deliberately chose video material that had been obtained in a natural task (free exploration) and for which we had previously established a relation between gaze orienting by head movements and Viola-Jones false alarms during the real-world situation [16]. Given this video material, the amount of usable misses (those that are an obvious face during prolonged viewing of the face *region*) seems too limited and too heterogeneous to be of reasonable use for the present experiment.

In contrast to the present study, which is only concerned with face *detection*, research on face processing often focuses on the *recognition* of faces. Such recognition could also be mediated by comparably simple features: for example, famous faces can be recognized based on horizontal structure (“bar codes” [18]). Akin to the susceptibility of face detection to rotation reported here, face recognition is affected by an inversion effect [19], which also depends on horizontal bars [20]. Although the neural substrate for detection and recognition of faces may overlap [21] - for example both involve the fusiform face area (FFA) - failure of recognition does not imply impairment of detection. In fact, recent studies on prosopagnosic patients suggest that face detection can be fully preserved, even when recognition is severely impaired [22], [23]. Consequently, it is not self-evident that results from recognition transfer to detection and vice-versa. In line with an earlier results in prosopagnosic patients and healthy controls [24], we here show explicitly that not only face recognition but also face detection depends on orientation. The identification of commonalities and differences between detection and recognition is crucial in understanding the visual processing hierarchy for faces, and other items.

The difference between manual responses and responses by shifts of gaze, renders it likely that Viola-Jones features are used primarily in a pre-attentive mode of detection. Given that even early phases of face processing interact with the task [25], Viola-Jones features may be recruited in particular when the task demands rapid processing, while more “conscious” processing is deferred to high levels, such as FFA [21]. A pre-attentive interpretation is in line with the usefulness of a Viola-Jones face “channel” in attention models [26], and the difference between coarse orienting by head movements, which is susceptible to illusory faces, and fine orienting by eye movements, which is not [16]. With oriented features as key to a first computation of face-likeness, the nearly unavoidable attention to faces [27], could be reconciled with an early (V1) representation of an attention-driving saliency map [28]. In similar fashion, face pop-out, which has been used as evidence for the involvement of high level areas in visual search [10], [12] (but see [11]), might originate in early visual areas. Alternatively, Viola-Jones-like filters may support a very rapid initial estimate of face location that is then refined for further processing. In this view, which is in line with the reverse-hierarchy theory [29], Viola-Jones-like processes need not to be tied to a specific early visual brain area, but can be at the foundation of the “gist” estimate that precedes fine-grain processing. Support for this view arises from a study in a prosopagnosic patient [23]. Despite lesions in early visual areas, including the occipital face area, this patient can detect Mooney faces with a performance in the range of healthy controls and shows activation of high-level visual areas, including the fusiform face area (FFA). Since the Viola-Jones algorithm also copes well with Mooney faces (figure S2), the “holistic” processing probed by Mooney faces and the fact that Viola-Jones is based on “features” are not in conflict. In fact, the primary Haar filter (figure 2g), i.e., the simplest “feature” of the Viola-Jones algorithm, well resembles a very coarse estimate of a face (a dark region horizontally sandwiched between two bright regions), which could readily be employed by higher-level visual area with a large receptive field. The reverse-hierarchy coarse-to-fine explanation is also consistent with a result from free exploration: in this real-world setting illusory faces only trigger an initial coarse orienting of gaze by head movements that then frequently is not refined by eye movements, if fine-grain scrutiny rejects the hypothesis of the illusory face being a real face [16]. Irrespective of whether Viola-Jones-like face processing happens in early visual cortex, in higher areas or both, the present results show that it is a viable model for an initial, pre-attentive, estimate in human face detection.

When observers are asked to make an eye movement as quickly as possible to the image of a face that is visible for 400 ms, ultra rapid saccades still go to the face stimulus when its phase is scrambled and no localized information is available [13]. This finding is, however, not in conflict with our present data. In our case the stimulus is shown briefly (20 ms) and masked thereafter, while in [13] the stimulus is visible when the saccade is executed. In fact, we do not observe ultra rapid saccades in our paradigm. This suggests that two distinct processes might be responsible for the ultra rapid saccades during unmasked and considerably long viewing presentation on the one hand and during detection in brief presentation and subsequent decision on the other hand. It is conceivable that the ultra rapid saccades include subcortical pathways thus bypassing visual cortex [1]. Indeed, it has been suggested that an innate face-detection system employs subcortical areas such as the superior colliculus and the pulvinar [5], [6]. In this view, ultra-rapid saccade paradigms recruit the subcortical system, while detection during brief presentation involves the oriented and localized filters of V1. Irrespective of whether the V1 hypothesis of human face detection will eventually prove true, our data clearly demonstrate that simple features, oriented localized luminance transitions are not only useful in computer vision, but involved in human face detection as well.

## Materials and Methods

### Observers and Ethics Statement

Twenty-four volunteers, recruited at the university, participated in the experiment, after giving written informed consent. Eight participated in the first experiment (age: 18–25; mean age: 21.0±2.1 years; 4 female), eight in the second experiment (age: 19–27; mean age: 23.1±2.5 years; 4 female) and eight in the third experiment (age: 22–29; mean age: 24.5±2.1 years; 4 female). Procedures were in accordance with national and institutional regulations as well as the Declaration of Helsinki and were approved by the Ethikkommission des Fachbereichs Psychologie der Philipps-Universität Marburg.

### Stimuli

All stimuli were gathered from a set of first person perspective movies [16] (recorded with the “EyeSeeCam” [30]). The Viola-Jones [14] algorithm with the default training set provided in OpenCV 1.0 [31] was applied to these movies. Detections of the algorithm were manually classified into real and illusory faces; face detections on posters, mannequins, statues and the like were discarded. To ensure that the stimuli were from near the focus of attention of the wearer of the eye-tracker, only detections from the central third in each direction were used. Further constraints were imposed for the use of a detection as stimulus: first, stimuli had to be the unique detection in their frame of the movie; second, no two stimuli could originate from frames spaced less than a second apart; third, detections used as stimuli had to be at least 50×50 pixels in size. To ensure the full face was visible in the stimulus, the detection plus 20% in each direction were cut (e.g., a 50×50 detection would yield 70×70 stimulus). Subsequently, all stimuli were downsampled to 70×70 pixels resolution using bicubic interpolation. Non face stimuli were picked at the same locations and sizes from frames that did not contain detections and using the same constraints as the other categories. For experiment 1 the first fifty stimuli of each category fulfilling these constraints were selected; stimuli for experiment 2 were selected as subset of those.

Masks were generated by computing the average power spectrum of all stimuli across all categories used in the respective experiment and assigning it with a random phase. A different mask (with different random phase) was used in each trial, but the same mask for both stimuli in each trial.

To assess the effects of luminance and luminance contrast, the screen's pixel value to luminance mapping (“gamma”) was measured for each gun and used to compute the actually presented stimulus luminance.

### Setup

An EyeLink 2000 (SR Research, Missasuaga MR, Canada) was used to record eye movements. Specifically, saccades were detected using the manufacturer's recommended settings (velocity threshold 35°/s, acceleration threshold 9,500°/s^2^). Stimuli were presented on an 19.7” EIZO FlexScan F77S CRT monitor at a distance of 48 cm from observers' eyes.

### Procedures

In all experiments, each trial started with the presentation of a green dot for 1 s. In all experiments, each trial started with the presentation of a green dot for at least 1 s. In experiments 1 and 2, the dot turned white and disappeared only after it was fixated; in experiment 3 the dot disappeared as soon as the 1 s had elapsed. After a further 300 ms two stimuli (14×14 degrees visual angle) were presented 2.3 degrees left and right of the fixation dot for 20 ms and followed by a mask. The mask stayed on for 500 ms after the observer made a saccade (experiments 1 and 2) or until the observer pressed a button (experiment 3). In experiments 1 and 2, observers were instructed to direct their gaze to the side where they thought the face had been as fast as possible. The direction of the first saccade (left or right) was used as the response. In experiment 3 a button press (left arrow/right arrow) replaced the eye-movement response. In all experiments, observers were instructed to blink only when the green fixation dot was presented. To prevent any learning, in particular of illusory faces as compared to non faces, no explicit feedback on the correctness of the response was given in any of the experiments. By masking the stimuli (see above), we are also confident that observers could not infer the correctness of their selection and thus no implicit feedback was available either.

For experiments 1 and 3, 1500 pairs of stimuli were generated, 500 from each pair of categories (real face vs. non face, real face vs. illusory face, illusory face vs. non face), such that each stimulus appeared in exactly 10 stimulus pairs per pair of categories. The order of pairs was randomized for each observer as was the position (left/right) of the stimuli. The pairs were presented in 15 blocks of 100 trials, with the possibility to take a break between blocks.

In experiment 2 ten real faces and ten false positives with average performance in experiment 1 were chosen. Each trial used a pair of a real face and an illusory face. The orientation of both the real and illusory face could be upright, or rotated 90° counterclockwise, making for 4 combinations of orientation. By presenting all possible combinations of real and illusory faces (100) in all 4 orientation combinations both in color and in grayscale, 800 (100×4×2) trials were generated. The order of pairs was randomized within observer, as was the position of the stimuli (left/right). Each observer either first did 4 blocks of 100 color pairs, then 4 blocks of 100 grayscale pairs, or vice versa, counterbalanced over observers.

## Supporting Information

Figure S1 **Visualization of the average illusory face.** To visualize how averaging illusory faces quickly leads to the impression of a face even during prolonged viewing, we sequentially averaged 2,3,…50 illusory face stimuli, once ordered by performance, once ordered randomly. **Top row**: 50 illusory faces sorted by the probability of being selected as face in experiment 1 (as figure 2B). **Second row**: n-th image depicts average (pixelwise arithmetic mean per color channel) of the n leftmost faces of top row. **Third row**: 50 illusory faces in random order. **Bottom row**: n-th image depicts average of the n leftmost faces of third row. In both scenarios, a small number of illusory face stimuli suffices to evoke the subjective impression of a face in the average stimulus.
(PDF)

Figure S2 **Mooney faces.** To test the holistic aspects of face processing against the use of individual features, face images are frequently reduced to half-tone (binary) images, consisting only of black and white areas, so-called Mooney faces [Mooney CM (1957) *Canad J Psychol* 11(4): 219–226]. Since human observers proficiently process these faces and detection is rarely problematic for upright stimuli, but detection degrades for rotated versions [Jeffreys DA (1993) *Exp Brain Res* 96:163–172], Mooney faces also seem to lend themselves to the purpose of the current study. Provided human proficiency with Mooney faces, failure of the algorithm to detect Mooney faces would be a strong argument against the similarity of algorithm and human. Hence we tested the algorithm with two versions of Mooney-like stimuli based on the 50 images from which the real-face stimuli were taken. In the first version, we were agnostic about the original face detection: we binarized the image by using the median of the image's gray values as a threshold, with everything brighter than the median colored white and everything darker colored black. By design, this procedure resulted in a Mooney-like image with about 50% of the area being white and the remaining about 50% being black. **Left panel**: In the majority (28/50) of these images, the Viola-Jones algorithm still correctly detected the face. **Right panel**: Of the 22/50 misses, many were a consequence of the whole-image median resulting in the face area being predominately of one color, and only very few of the face-containing patches are readily discernible as face for humans even during prolonged viewing. If – in a second version of the stimuli - the threshold is based on the median gray value of the face region rather than the image, Viola-Jones detection succeeds in 88% (44/50) of the cases. These data show that – like humans – the Viola-Jones algorithm can detect most Mooney faces, and furthermore misses happen for images that qualitatively seem difficult for human observers, too. In retrospect this is rather unsurprising, as the features used by the Viola-Jones algorithm are mostly preserved in the conversion to Mooney faces. Nonetheless, this results somewhat strengthens the similarity of Viola-Jones and human face detection, but also implies that testing human observers on Mooney faces will provide little additional insight in the present context of comparing the Viola-Jones algorithm to human performance.
(PDF)
