# Supplementary material for: Faces in Places: Humans and Machines Make Similar Face Detection Errors
Source: PLoS One. 2011 Oct 5;6(10):e25373. doi: 10.1371/journal.pone.0025373 (PMC3187842; doi:10.1371/journal.pone.0025373)
Supplement: Figure S1 — Visualization of the average illusory face. To visualize how averaging illusory faces quickly leads to the impression of a face even during prolonged viewing, we sequentially averaged 2,3,…50 illusory face stimuli, once ordered by performance, once ordered randomly. Top row: 50 illusory faces sorted by the probability of being selected as face in experiment 1 (as figure 2B). Second row: n-th image depicts average (pixelwise arithmetic mean per color channel) of the n leftmost faces of top row. Third row: 50 illusory faces in random order. Bottom row: n-th image depicts average of the n leftmost faces of third row. In both scenarios, a small number of illusory face stimuli suffices to evoke the subjective impression of a face in the average stimulus. (PDF) [file pone.0025373.s001.pdf]

Figure S1 - Visualization of the average illusory face

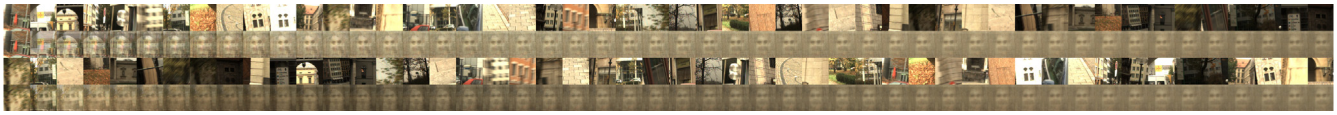

To visualize how averaging illusory faces quickly leads to the impression of a face even during prolonged viewing, we sequentially averaged 2,3,…50 illusory face stimuli, once ordered by performance, once ordered randomly. ***Top row****: 50 illusory faces sorted by the probability of being selected as face in experiment 1 (as figure 2B).* ***Second row****: n-th image depicts average (pixelwise arithmetic mean per color channel) of the n leftmost faces of top row.* ***Third row****: 50 illusory faces in random order.* ***Bottom row****: n-th image depicts average of the n leftmost faces of third row. In both scenarios, a small number of illusory face stimuli suffices to evoke the subjective impression of a face in the average stimulus.*
