# Supplementary material for: Faces in Places: Humans and Machines Make Similar Face Detection Errors
Source: PLoS One. 2011 Oct 5;6(10):e25373. doi: 10.1371/journal.pone.0025373 (PMC3187842; doi:10.1371/journal.pone.0025373)
Supplement: Figure S2 — Mooney faces. To test the holistic aspects of face processing against the use of individual features, face images are frequently reduced to half-tone (binary) images, consisting only of black and white areas, so-called Mooney faces [Mooney CM (1957) Canad J Psychol 11(4): 219–226]. Since human observers proficiently process these faces and detection is rarely problematic for upright stimuli, but detection degrades for rotated versions [Jeffreys DA (1993) Exp Brain Res 96:163–172], Mooney faces also seem to lend themselves to the purpose of the current study. Provided human proficiency with Mooney faces, failure of the algorithm to detect Mooney faces would be a strong argument against the similarity of algorithm and human. Hence we tested the algorithm with two versions of Mooney-like stimuli based on the 50 images from which the real-face stimuli were taken. In the first version, we were agnostic about the original face detection: we binarized the image by using the median of the image's gray values as a threshold, with everything brighter than the median colored white and everything darker colored black. By design, this procedure resulted in a Mooney-like image with about 50% of the area being white and the remaining about 50% being black. Left panel: In the majority (28/50) of these images, the Viola-Jones algorithm still correctly detected the face. Right panel: Of the 22/50 misses, many were a consequence of the whole-image median resulting in the face area being predominately of one color, and only very few of the face-containing patches are readily discernible as face for humans even during prolonged viewing. If – in a second version of the stimuli - the threshold is based on the median gray value of the face region rather than the image, Viola-Jones detection succeeds in 88% (44/50) of the cases. These data show that – like humans – the Viola-Jones algorithm can detect most Mooney faces, and furthermore misses happen for images that qualitatively seem diffic [file pone.0025373.s002.pdf]

Figure S2 - Mooney faces

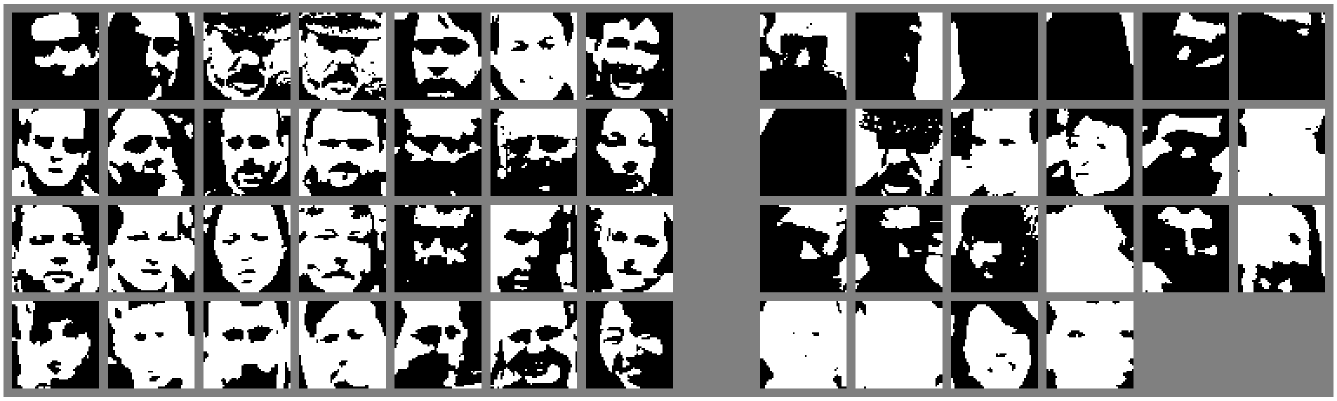

To test the holistic aspects of face processing against the use of individual features, face images are frequently reduced to half-tone (binary) images, consisting only of black and white areas, so-called Mooney faces [Mooney CM (1957) Canad J Psychol 11(4): 219-226]. Since human observers proficiently process these faces and detection is rarely problematic for upright stimuli, but detection degrades for rotated versions [Jeffreys DA (1993) Exp Brain Res 96:163-172], Mooney faces also seem to lend themselves to the purpose of the current study. Provided human proficiency with Mooney faces, failure of the algorithm to detect Mooney faces would be a strong argument against the similarity of algorithm and human. Hence we tested the algorithm with two versions of Mooney-like stimuli based on the 50 images from which the real-face stimuli were taken. In the first version, we were agnostic about the original face detection: we binarized the image by using the median of the image's gray values as a threshold, with everything brighter than the median colored white and everything darker colored black. By design, this procedure resulted in a Mooney-like image with about 50% of the area being white and the remaining about 50% being black. ***Left panel:*** *In the majority (28/50) of these images, the Viola-Jones algorithm still correctly detected the face.* ***Right panel:*** *Of the 22/50 misses, many were a consequence of the whole-image median resulting in the face area being predominately of one color, and only very few of the face-containing patches are readily discernible as face for humans even during prolonged viewing. If – in a second version of the stimuli - the threshold is based on the median gray value of the face region rather than the image, Viola-Jones detection succeeds in 88% (44/50) of the cases. These data show that – like humans – the Viola-Jones algorithm can detect most Mooney faces, and furthermore misses happen for images that qualitatively seem difficult for human observers, too. In retrospect this is rather unsurprising, as the features used by the Viola-Jones algorithm are mostly preserved in the conversion to Mooney faces. Nonetheless, this results somewhat strengthens the similarity of Viola-Jones and human face detection, but also implies that testing human observers on Mooney faces will provide little additional insight in the present context of comparing the Viola-Jones algorithm to human performance.*
